# Pioglitazone versus Rosiglitazone: Effects on Lipids, Lipoproteins, and Apolipoproteins in Head-to-Head Randomized Clinical Studies

**DOI:** 10.1155/2008/520465

**Published:** 2008-08-21

**Authors:** Mark A. Deeg, Meng H. Tan

**Affiliations:** Endocrine Research and Clinical Investigation, Lilly Research Laboratories, Eli Lilly & Co., Indianapolis, IN 46285, USA

## Abstract

Peroxisome proliferator-activated receptors (PPARs) play an important role in regulating both glucose and lipid metabolism. Agonists for both PPAR*γ* and PPAR*γ* have been used to treat dyslipidemia and hyperglycemia, respectively. In addition to affecting glucose metabolism, PPAR*γ* agonists also regulate lipid metabolism. In this review, we will focus on the randomized clinical trials that directly compared the lipid effects of the thiazolidinedione class of PPAR*γ* agonists, pioglitazone and rosiglitazone, head-to-head either as monotherapy or in combination with other lipid-altering or glucose-lowering agents

## 1. INTRODUCTION

Peroxisome proliferator-activated receptors (PPARs) play an important role in regulating both glucose and lipid metabolism. Agonists for both PPAR*α* and PPAR*γ* have been used to treat dyslipidemia and hyperglycemia, respectively. In addition to affecting glucose metabolism, PPAR*γ* agonists also regulate lipid metabolism.

The dyslipidemia of type 2 diabetes mellitus is characterized by elevations in serum triglycerides and increased very low-density lipoprotein (VLDL) particle size, reduced high-density lipoprotein (HDL) cholesterol and HDL particle size, and the predominance of small, dense low-density lipoprotein (LDL) particles with generally normal LDL cholesterol. Many studies have examined the effect of improvements in glycemic control on serum lipids and lipoproteins utilizing a variety of glucose-lowering medications [1]. These include insulin, sulfonylureas, biguanides, thiazolidinediones, glucagon-like peptides, *α*-glucosidase inhibitors, and dipeptidyl peptidase-IV inhibitors. In general, improving glycemic control reduces serum triglycerides and increases HDL cholesterol. Numerous studies have compared the effect of thiazolidinediones with other oral glucose-lowering medications. In general, thiazolidinediones have better overall effects on lipids compared to sulfonylureas or insulin [2, 3]. In this review, we will focus on the randomized clinical trials that directly compared the lipid effects of the thiazolidinedione class of PPAR*γ* agonists, pioglitazone and rosiglitazone, head to head either as monotherapy or in combination with other lipid-altering or glucose-lowering agents. The effects of troglitazone (Rezulin), which has been removed from the market, will not be discussed.

## 2. ROLE OF PPAR*γ* IN REGULATING FATTY ACID/TRIGLYCERIDE METABOLISM

**Figure 1:**
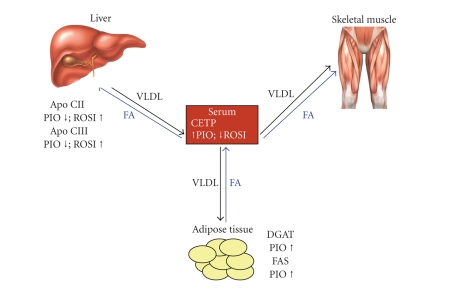


The whole-body response to activating PPAR*γ* is storage of energy, as triglycerides, in adipocytes. This is accomplished by the coordinated regulation of tissue-specific gene expression in adipocytes, liver, and cells that utilize fatty acids for energy as well as various circulating factors that coordinate and regulate fatty acid synthesis and utilization. Although often only serum triglycerides are measured and monitored in patients, serum triglycerides represent just one compartment within which PPAR*γ* medications affect whole-body triglyceride/fatty acid metabolism. Serum triglycerides within VLDL and chylomicrons may be considered the mechanism by which energy (as triglycerides) is transported from one tissue to another (Figure 1).

In the adipocyte, both pioglitazone and rosiglitazone increase the expression of genes associated with hydrolysis of triglyceride-rich lipoproteins and fatty acid uptake and storage [4, 5] (Figure 1). Thiazolidinediones also reduce fatty acid release from adipocytes. This in turn leads to less fatty acid delivery to the liver and a decrease in hepatic triglyceride synthesis. In addition, PPAR*γ* medications influence secretion of adipokines that affect lipid and glucose metabolism. Pioglitazone and rosiglitazone therapies increase adiponectin [6, 7] and decrease retinol binding protein 4 [8] and resistin [9]. These adipokines influence lipid metabolism and insulin sensitivity.

In the liver, PPAR*γ* therapy is associated with changes in expression of various genes involved in lipid metabolism including apolipoproteins CII and CIII. Apolipoproteins CII and CIII stimulate and inhibit lipoprotein lipase, respectively. Lipoprotein lipase is the major enzyme involved in hydrolyzing and removing triglyceride-rich lipoproteins from the serum.

## 3. COMPARISON OF LIPID EFFECTS OF PIOGLITAZONE AND ROSIGLITAZONE IN HEAD-TO-HEAD RANDOMIZED CLINICAL TRIALS

### 3.1. Thiazolidinediones as monotherapy: effects on fasting lipids

Goldberg et al. [10] and Deeg et al. [11] compared the effects of pioglitazone and rosiglitazone in patients with type 2 diabetes mellitus and dyslipidemia on non-lipid-altering medications (see Table 1). After discontinuing their glucose-lowering and lipid-altering medications, if they were on them, patients were randomized to pioglitazone or rosiglitazone. Patients were treated with 30 mg once a day (QD) of pioglitazone or 4 mg of rosiglitazone QD for 12 weeks with a forced titration to 45 mg QD and 4 mg twice a day (bid) for additional 12 weeks, respectively. Both medications reduced hemoglobin A1c (A1c), insulin resistance (as determined by HOMA-IR), and fasting free fatty acids to a similar extent. However, the effects on fasting triglycerides were divergent. Pioglitazone therapy was associated with a reduction in fasting triglycerides throughout the study, whereas rosiglitazone increased triglycerides within 4 weeks, which then declined with time. At the end of the study, triglycerides were decreased by 12% with pioglitazone, and elevated by 15% in patients on rosiglitazone.

The decrease in triglycerides with pioglitazone was associated with a decrease in large VLDL and intermediate density lipoproteins (IDLs), whereas the increase in triglycerides with rosiglitazone was associated with an increase in both large- and medium-sized VLDL and IDL concentrations. Pioglitazone decreased whereas rosiglitazone increased apolipoprotein CIII.

Both medications raised LDL cholesterol; however, the increase was significantly greater with rosiglitazone compared to pioglitazone (12.3% and 21.3%, resp.). Both therapies increased the average size of LDL particles, but the effect of pioglitazone was greater than that of rosiglitazone. Consistent with the changes in LDL cholesterol, pioglitazone did not significantly change apolipoprotein B levels but did reduce LDL particle concentration. Conversely, rosiglitazone increased both apolipoprotein B and LDL particle concentration. The clinical significance of the difference in particle concentration is unclear although decreased LDL particle concentration has been associated with a reduced risk for coronary heart disease [12, 13]. Both medications raised serum levels of lipoprotein (a).

As expected, both medications increased HDL cholesterol and the average size of HDL particles; however the increase in HDL cholesterol was significantly greater with pioglitazone therapy compared with rosiglitazone therapy (14.9% and 7.8%, resp.). Again, there was a difference in HDL particle subclasses between the medications. Pioglitazone increased total, large, and medium HDLs while decreasing small HDL concentration. Rosiglitazone, in contrast, decreased total, large, and small HDLs while increasing medium HDL particle concentration. These suggest that there are differences in HDL metabolism with these two agents. Pioglitazone had no effect on serum apolipoprotein AI levels, but rosiglitazone therapy was associated with a decrease in apolipoprotein AI levels.

### 3.2. Thiazolidinediones as monotherapy: effects on postprandial lipemia

Postprandial dyslipidemia is a feature of type 2 diabetes. Two small studies compared the effects of pioglitazone and rosiglitazone on postprandial lipemia using a prospective, randomized crossover design [14, 15]. After washing out both glucose-lowering (8 weeks) and lipid-altering medications (4 weeks), patients were randomized to either pioglitazone (30 mg QD for 4 weeks, then 45 mg QD for 8 weeks) or rosiglitazone (4 mg QD for 4 weeks followed by 4 mg bid for 8 weeks) with an 8-week washout during the crossover. Before and after each treatment, a standardized breakfast was served and postprandial glucose, lipids, and hormones were measured.

Both agents had similar effects on A1c and HOMA-IR. Pioglitazone reduced fasting and postprandial triglycerides that were associated with decreases in the smaller VLDL subfractions: VLDL-2 and VLDL-3. Rosiglitazone increased the postprandial triglycerides with increases in VLDL-2 and VLDL-3. There was no effect with either medication on fasting apolipoprotein B, AI, or CII/CIII ratio, and lipoprotein lipase or hepatic lipase activity did not differ between therapies. Cholesterol ester transfer protein activity decreased with rosiglitazone and increased after pioglitazone therapy. The second study demonstrated that pioglitazone was more effective than rosiglitazone in increasing larger LDL concentrations (fasting and postprandial) as well as in reducing levels of small, dense LDL particles [14].

### 3.3. Thiazolidinediones in combination with other oral antihyperglycemic medications

Derosa et al. [16] compared the effect of adding pioglitazone (15 mg QD) or rosiglitazone (4 mg QD) on patients with type 2 diabetes treated with glimepiride (4 mg QD). After 12 months, both groups had significant reductions in A1c (1.3%). The group treated with the pioglitazone combination had a reduction in total cholesterol, LDL cholesterol, lipoprotein (a), and apolipoprotein B with an increase in HDL cholesterol. The rosiglitazone group had increases in total cholesterol, LDL cholesterol, triglycerides, and apolipoprotein B but no effect on HDL cholesterol or lipoprotein (a) [17]. Both groups showed a reduction in homocysteine.

In a similarly designed trial, patients with type 2 diabetes were treated with metformin and randomized to pioglitazone or rosiglitazone [18]. After 12 months, both groups had similar reductions in A1c and insulin resistance (as determined by HOMA-IR). Total cholesterol, LDL cholesterol, triglycerides, and apolipoprotein B decreased in the pioglitazone group with increases in HDL cholesterol and apolipoprotein AI. There were no changes observed in the rosiglitazone group.

### 3.4. Thiazolidinediones in combination with statins

Berhanu et al. [19] examined the changes in lipids when patients were switched from rosiglitazone and a statin to pioglitazone (30 mg) while maintaining a stable statin dose. At the end of the trial (17 weeks), although the A1c did not change, patients had a significant reduction in triglycerides, total cholesterol, and LDL particle concentration (189 nmol/L) and increases in LDL cholesterol, HDL cholesterol, and LDL particle diameter (0.23 nm). Apolipoprotein B did not change but apolipoprotein AI increased.

In summary, although the head-to-head and rosiglitazone-only [20] clinical trials demonstrate a benefit of rosiglitazone on HDL cholesterol, there isa relatively consistent and overall favorable impact of pioglitazone compared to rosiglitazone on serum lipids, lipoproteins, and apolipoproteins. It is also clear that the lipids' effects are unrelated to the changes in insulin sensitivity since [1] both agents have similar effects to improve insulin sensitivity and [2] the effect on insulin sensitivity can be clearly differentiated from lipid changes [21]. Thus, there must be other differences in the action of the thiazolidinediones that account for the divergent lipid effects.

### 3.5. Comparison of mechanisms of action on lipid metabolism

Whole-body fatty acid/triglyceride metabolism involves the interaction of numerous organs as described above. Since both pioglitazone and rosiglitazone have similar effects in the adipocyte on adipokines' expression and genes involved in fatty acid/triglyceride metabolism, the difference between these medications on serum triglycerides likely occurs within the liver and/or plasma compartment.

The most profound difference between the lipid effects of pioglitazone versus rosiglitazone is in fasting and postprandial triglycerides. As both medications have similar effects on glycemic control and insulin resistance, an additional mechanism must account for these differences. The differences in serum triglycerides occur in smaller VLDL particles which are produced in an insulin-independent fashion consistent with the observations that it is not the change in insulin resistance that accounts for the differences. One potential difference, which may account for the difference, is the effect on apolipoprotein CIII. Two studies have demonstrated that pioglitazone decreases and rosiglitazone increases apolipoprotein CIII [10, 22]. A decrease in apolipoprotein CIII would lead to an increase in lipoprotein lipase activity, and hence an increase in the hydrolysis of triglycerides and catabolic rate of triglyceride-rich lipoproteins including chylomicrons and VLDL [23]. This hypothesis is supported by the observation that pioglitazone increases the lipolysis of VLDL triglycerides without affecting the removal of VLDL particles [22]. Conversely, rosiglitazone increases the production and reduces the catabolism of triglyceride-rich lipoproteins including both VLDL and chylomicrons [21].

Another possibility is that genetic differences may contribute to the different lipid effects. Polymorphism of the PPAR*γ*2 gene influences the glycemic response to rosiglitazone [24] but not to pioglitazone [25]. A lipoprotein lipase variant influences the glycemic effect of pioglitazone [26], while a polymorphism of the adiponectin [27] and perilipin [28] genes influences the glycemic and weight gain responses, respectively, to rosiglitazone. Since none of these studies directly compared both rosiglitazone and pioglitazone, it is unclear if polymorphism contributes to the differences. Most of these studies also did not show a linkage between lipid effects and polymorphisms, but a link between the adiponectin genotype at position 45 and the triglyceride effect of rosiglitazone did statistically approach significance [27]. Whether this occurs with pioglitazone has not been published to date.

It is possible that pharmacokinetic differences between pioglitazone and rosiglitazone may account for the differences in lipid effects; however, this is an unlikely contributor since the gene expression and pharmacodynamic effects of both agents exceed the presence of active drug in the serum.

Do the differences in lipid effects have clinical significance? Increased fasting and postprandial triglycerides [29, 30] as well as LDL particle concentration [12, 13] are risk factors for cardiovascular disease. Conversely, increases in large HDL and adiponectin are associated with reduction in risk. It is also likely that other effects influence the risk of coronary artery disease (CAD) events. It is likely that the integrated sum of these lipid effects, together with yet-defined factors, will determine the influence on atherosclerosis.

Clinical outcome trials with both pioglitazone and rosiglitazone have been published. Both pioglitazone and rosiglitazone improve endothelial function and reduce the progression of carotid intramedial thickness in patients [31–34]. These observations suggest a clinical benefit with both agents. In the PROACTIVE study, adding pioglitazone to the current treatment in patients with type 2 diabetes was associated with reductions in major atherosclerotic events as defined in the main secondary end-point [35], recurrent myocardial infarction [36], and recurrent stroke [37]. Meta-analysis of pioglitazone clinical trials showed a significantly lower risk of death, myocardial infarction, or stroke in patients with diabetes [20].

The effect of rosiglitazone on CAD events is more controversial. Some post hoc meta-analysis studies have suggested that rosiglitazone is associated with an increased risk of CAD events [38, 39]. However, in the RECORD trial, a prospective trial in patients with type 2 diabetes, no evidence for an increased event rate was found in an interim analysis [40]. Completion of this along with other studies is needed to fully answer the effect of rosiglitazone on CAD events.

## 4. SUMMARY

Both pioglitazone and rosiglitazone reduce insulin resistance and improve glycemic control in patients with type 2 diabetes. However, the head-to-head clinical trials demonstrate a relatively consistent and favorable impact of pioglitazone compared to rosiglitazone on serum lipids, lipoproteins, and apolipoproteins. Whether these differences result in different outcomes that are clinically significant remains to be determined.

## Figures and Tables

**Table 1:** Summary of clinical trials comparing lipid effects of pioglitazone and rosiglitazone.

	Concomitant glucose/lipid therapy	*N*	Duration	Pioglitazone effects	Rosiglitazone effects
Derosa et al. [16, 17]	Glimepiride	91	52 wk	↑HDL-C, apo AI	↑TC*, LDL-C*, HDL-C,
				↓TC*, LDL-C*,	↑TG*, apo AI, apo B*, lipoprotein (a)
				↓TG*, apo B*, Lp(a)*	

Goldberg et al. [10], Deeg et al. [11]	None	802	24 wk	↑HDL-C*, LDL-C*, TC*	↑TG*, HDL-C*, LDL-C*,
				↓TG*	↑TC*, apo B*
				↑VLDL-P, HDL-P*, apo AI	↑VLDL-P, HDL-P*, LDL-P*, apo CIII*
				↓LDL-P*, apo CIII*	↓apo AI*

Berhanu et al. [19]	Statins	305	17 wk	↓TG*, TC*, LDL-P,
				↑LDL-C*, HDL-C
				(changes following switch from rosiglitazone to pioglitazone)

Chappuis et al. [15]	None	17	12 wk	↓AUC-TG*	↑AUC-TG*
				↑CETP*	↓CETP*

Derosa et al. [18]	Metformin	96	52 wk	↓TC*, LDL-C*, TG*, apo B*	No significant changes in any lipid parameter
				↑HDL-C*, apo AI*	

Berneis et al. [14]	None	9	12 wk	↑TC, HDL, LDL, LDL IIA*	↑TC, TG*, HDL, LDL, LDL-IIA
				↓TG*	

*N* = number of patients enrolled. Pioglitazone and rosiglitazone effects are summarized as % change from baseline and listed in parentheses. (*) indicates a statistically significant change from baseline. TC = total cholesterol, TG = triglycerides, LDL-C = LDL cholesterol, HDL-C = HDL cholesterol, LDL-P = LDL particle number, HDL-P = HDL particle number, apo = apolipoprotein, AUC-TG = area under the curve for TG.
